# A study protocol for the modified interactive screening program plus MINDBODYSTRONG^©^ RCT: A mental health resiliency intervention for nurses

**DOI:** 10.1371/journal.pone.0303425

**Published:** 2024-06-06

**Authors:** Bernadette Mazurek Melnyk, Judy E. Davidson, Cora Mayfield, Sidney Zisook, Sharon Tucker, Andreanna Pavan Hsieh, Andrea Cooper, Rosalind Gray-Bauer, Jacqueline Hoying, Alison F. Cuccia, Alai Tan

**Affiliations:** 1 Vice President for Health Promotion, The Ohio State University, Columbus, Ohio, United States of America; 2 Office of the Chief Wellness Officer, The Ohio State University, Columbus, Ohio, United States of America; 3 Helene Fuld Health Trust National Institute for Evidence-Based Practice, The Ohio State University, Columbus, Ohio, United States of America; 4 College of Nursing, The Ohio State University, Columbus, Ohio, United States of America; 5 University of San Diego Health, San Diego, California, United States of America; 6 Nursing Programs, American Nurses Association Enterprise, Silver Spring, Maryland, United States of America; University of São Paulo, BRAZIL

## Abstract

**Background:**

Nurses, the largest workforce in healthcare, are at high risk of depression, anxiety, burnout, and suicidal ideation. Suicide among nurses is higher than the general population. This randomized controlled trial pairs the MINDBODYSTRONG^©^ cognitive-behavioral skills building program with the American Foundation for Suicide Prevention’s (AFSP) Modified Interactive Screening Program (mISP) to reduce depression, suicidal ideation, post-traumatic stress, anxiety, and burnout, and improve healthy lifestyle beliefs, healthy lifestyle behaviors, and job satisfaction in nurses with moderate to high risk of suicide.

**Aims:**

This study aims to determine the effects of the mISP combined with the digitized MINDBODYSTRONG^©^ program versus the mISP alone on depression, suicidal ideation, burnout, anxiety, post-traumatic stress, healthy lifestyle beliefs, healthy lifestyle behaviors, and job satisfaction in 364 U.S. nurses.

**Methods:**

A digitized version of MINDBODYSTRONG^©^ combined with the mISP screening and referral platform will be compared to the AFSP mISP alone through a two-arm randomized controlled trial. Follow-up post-intervention data will be collected at week eight and months three, six, and 12.

**Discussion:**

If successful, this study’s findings could assist nurses who are hesitant to use conventional mental health resources by providing them with confidential aid and learning opportunities to reduce suicidality, depression, anxiety, post-traumatic stress, and burnout and improve healthy lifestyle beliefs, healthy lifestyle behaviors, and job satisfaction.

**Trial/study registration:**

The Ohio State University Protocol Record 2021B0417, Modified Interactive Screening Program Plus MINDBODYSTRONG: A Mental Health Resiliency Intervention for Nurses, is registered and posted at ClinicalTrials.gov Identifier: NCT05582343. First posted date is October 17, 2022.

## Introduction

Suicide remains a leading cause of death in the United States, with non-lethal suicide attempts as high as 10 to 20 times that of completed suicide attempts [1]. Both male and female nurses have a higher death by suicide rate than the general population [2]. The five million plus nurses that make up the largest healthcare workforce in the country report high levels of stress, burnout, and depression [3, 5]. The COVID-19 pandemic exacerbated these experiences and negatively impacted the mental and physical health of nurses [4–7]. A recent study of COVID-19 front-line nurses found that 65% were experiencing burnout [5]. Meanwhile, a systematic review with 16 studies and 18,935 nurses reported the prevalence of burnout via three Maslach Burnout Inventory subscales to be 34.1% emotional exhaustion, 12.6% depersonalization, and 15.2% lack of personal accomplishment [8]. The pandemic’s toll on the nursing profession has resulted in many leaving their jobs or the field entirely, resulting in the great resignation [9–11].

More than 70% of people who die by suicide do not receive needed services within the two months preceding their death [12], and only a small percentage of affected nurses receive needed mental health services [4, 13]. The stigma of seeking help for mental health touches the nursing profession in a significant way. Recent studies have found that nurses are concerned about the way seeking help can affect their employer’s perception of their mental fitness as well as concerns regarding licensure [14]. With 74% of states asking nurses invasive mental health questions on their licensure applications, the stigma continues to be a challenge [15]. Additionally, there is a shortage of mental health providers throughout much of the United States, which makes it difficult to receive timely care for those who pursue services [12].

This innovative study will be the first to assess a novel combination of the American Foundation for Suicide Prevention’s (AFSP) Interactive Screening Program (ISP) with an online digitized adaptation of the well-established manualized cognitive-behavioral skills building (CBSB) intervention, MINDBODYSTRONG^©^ (also known as Creating Opportunities for Personal Empowerment^©^ [COPE^©^]) in the literature), which has demonstrated efficacy in reducing depression, anxiety, and suicidal ideation as well as improving healthy lifestyle behaviors in children, teens, college students and clinicians [16–24].

AFSP’s ISP is a vendor-hosted, anonymous, and widely used mental health screening platform that identifies users with moderate to high risk for suicide and refers them to a therapist who assists in managing their urgent concerns and finding appropriate local resources [25–32]. Participating organizations purchase access to the encrypted, de-identified service and must staff their own mental health specialist to review screening outcomes and communicate with the users. It is endorsed and recognized as an evidence-based suicide prevention intervention and resource by the Council for Graduate Medical Education’s Tools and Resources for Physician Well-Being, American Hospital Association, and U.S. Surgeon General [33]. Use of the ISP has been studied in physicians, medical students, and nurses [25–32], but not in combination with a CBT skills-building program like MINDBODYSTRONG^©^. As done here, the ISP platform can be modified to address resources for specific populations directly; hereafter, it will be referred to as modified ISP (mISP). While AFSP hosts the secure, off-sight server for the screening and communication platform, they have no role in the design, data collection and analysis, decision to publish, or preparation of the manuscript for this protocol and study.

MINDBODYSTRONG^©^ has been adapted for nurses and other healthcare clinicians, with prior RCTs demonstrating significant improvements in participants’ depressive symptoms and job satisfaction [23, 24]. The original version of the program consists of seven manualized sessions that can be delivered by both non-mental health as well as psychiatric mental health professionals, making it more feasible for wide-scale implementation to nurses in healthcare systems across the U.S. Because 55% to 73% of nurses have experienced post-traumatic stress related to the COVID-19 pandemic [9, 34, 35], MINDBODYSTRONG^©^ also includes an optional added module on trauma that participants can choose to complete for a total of eight weekly sessions. Further, for ease of delivery across the nation, MINDBODYSTRONG^©^ has been digitized for this proposed protocol. MINDBODYSTRONG^©^ was selected as the cognitive-behavioral skills building programs of interest as, to our knowledge, other cognitive-behavioral skills building programs were not specifically developed for nurses and have not been tested with them, nor have any other programs been combined with the ISP screen.

Leveraging the strength of both the MINDBODYSTRONG^©^ and mISP could support better identification of suicide risk and offer more actionable support for nurses, potentially lessening mental health stigma and increasing access to confidential mental health care for nurses at risk for suicide.

It is hypothesized that nurses who receive the mISP screening and referral program in combination with the MINDBODYSTRONG^©^ CBSB program will report less depression, SI, post-traumatic stress, anxiety, burnout, and greater increases in personal beliefs, healthy lifestyle behaviors, and job satisfaction immediately post-intervention (8 weeks) as well as at the 3, 6, and 12 month post-intervention follow-up when compared to nurses who receive the mISP screening and referral program alone.

## Materials and methods

### Study aim

This study aims to determine the effects of mISP combined with the digitized MINDBODYSTRONG^©^ versus the mISP alone on depression, suicidal ideation, burnout, anxiety, post-traumatic stress, healthy lifestyle beliefs, healthy lifestyle behaviors, and job satisfaction in U.S. nurses.

### Study design

This study utilizes a two-arm, randomized controlled trial (RCT) design to determine the effects of mISP combined with digitized MINDBODYSTRONG^©^ verses mISP alone. The SPIRIT 2013 Checklist was followed (S1 File), and the approved IRB protocol can be viewed for more detail (S2 File). The mISP and MINDBODYSTRONG^©^ are carried out individually by the participant. Nurses identified with moderate to high risk of suicide through the mISP will be randomly assigned to MINDBODYSTRONG^©^ or the attention control group (i.e., mISP alone). Depression, suicidal ideation, burnout, anxiety, post-traumatic stress, healthy lifestyle beliefs, healthy lifestyle behaviors, and job satisfaction in U.S. nurses will be assessed to determine effectiveness. The post-intervention follow-up will occur at 8 weeks, 3, 6, and 12 months. These touchpoints for follow-up were selected to assess the sustainability of the intervention’s effect over time, determine whether a booster session would be needed during the first year, and identify what month would be most appropriate to do so.

**Fig 1:**
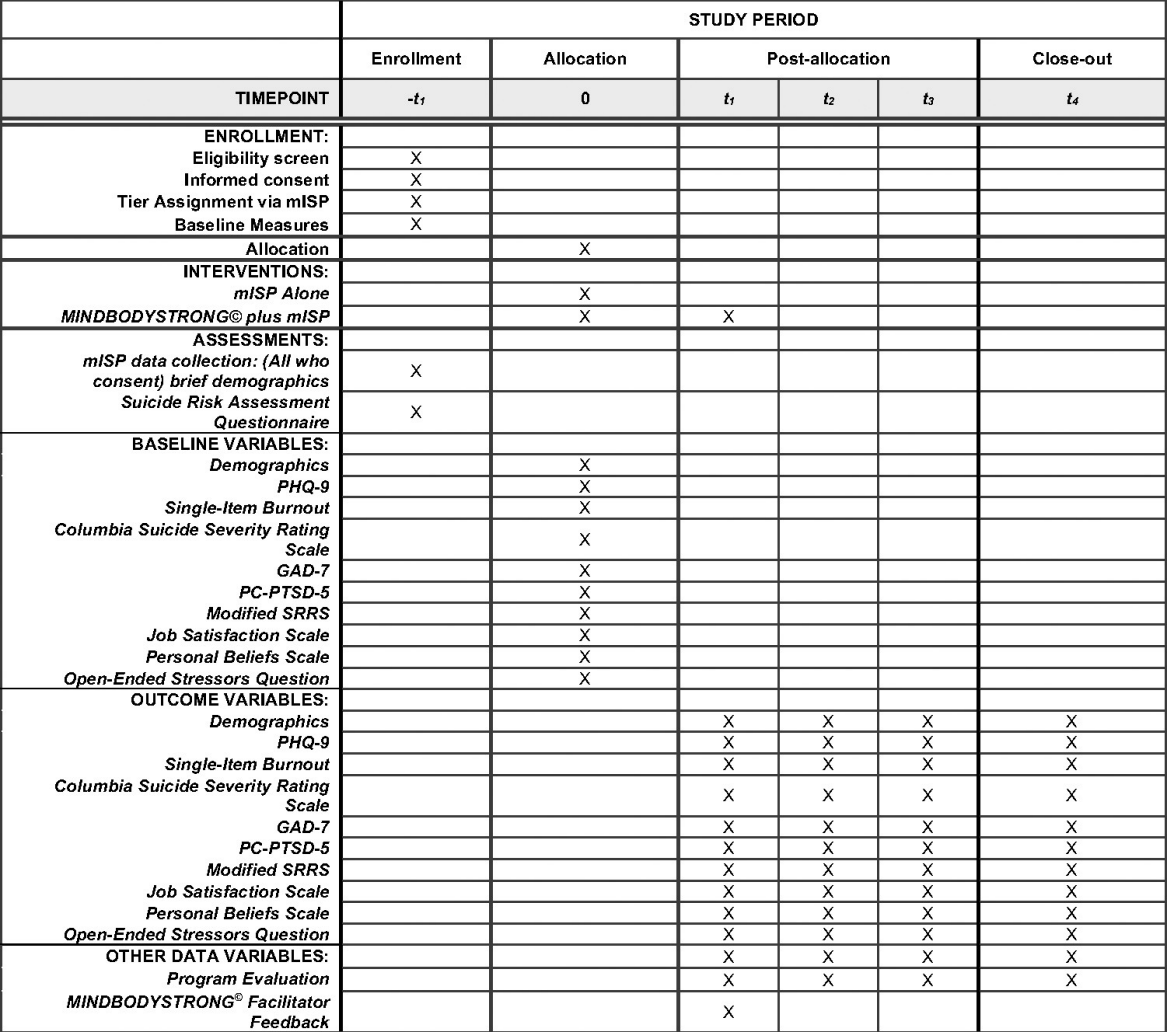
Schedule of enrollment, interventions, and assessments for mISP plus MINDBODYSTRONG^©^. Timepoints: -t0: Data collected after consent to determine eligibility, t1: baseline data collected at time of allocation, t1: 8 weeks (immediately post-intervention), t2: 3 months, t3: 6 months, t4: 12 months. mISP = Modified Interactive Screening Program; GAD-7 = Generalized Anxiety Disorder-7; PHQ-9 = Patient Health Questionnaire-9; PC-PTSD-5 = Primary Care Post Traumatic Stress Disorder Screen for Diagnostic and Statistical Manual of Mental Disorders-5; and SRRS = Social Readjustment Rating Scale. Specific Outcome variables from AIMs: depression, suicidal ideation, burnout, anxiety, post-traumatic stress, healthy lifestyle beliefs, healthy lifestyle behaviors, and job satisfaction.

Data will be collected on two platforms: (1) the mISP and (2) REDCap–a browser-based software specifically designed for clinical and translational research. Two platforms for data collection are required as the mISP is limited in its ability to link data overtime due to the encryption. To maintain data fidelity, an additional baseline survey will be completed by the participants in REDCap after completing the initial mISP screening, and all follow-up surveys will be taken in REDcap to allow for data linking at each point for analysis. Randomization will occur via REDCap. The schedule for enrollment, interventions, and assessments are viewable in Fig 1.

### Sample size and power analysis

A power analysis was conducted based on two-sample t-tests to compare pre- vs. postintervention means with two-tailed tests and a study-wide type I error rate of 5% to adjust for multiple comparisons from seven outcomes and four between-group pre-post comparisons per outcome. Given previous positive results with MINDBODYSTRONG©, yielding a large effect size of .98 requires a sample size of 30 (21 after accounting for 30% attrition), which would detect effects with 80%. However, given the untested nature of the electronic on-line version of MINDBODYSTRONG©, a medium effect of .5 was used to calculate a desired sample of 254, which would detect intervention effects on outcomes (depression, SI, burnout, anxiety, post-traumatic stress, healthy lifestyle beliefs, healthy lifestyle behaviors, and job satisfaction) with 80% power and study-wide type I error rate of 5%. With an expected 30% attrition, we will need a target recruitment of at least 364 nurses from the ANA to ensure an analytical sample size of 254.

### Sample and setting

The sample will primarily be nurse members of Healthy Nurse Healthy Nation (HNHN), a healthy lifestyle program through the American Nurses Association Enterprise (ANA). Healthy Nurse, Healthy Nation is a free online program designed for nurses to support six domains of wellbeing, including mental health. A membership with the ANA is not needed to join HNHN. The minimum sample size is 364 with 182 participants per arm. To reach this sample size, a random sample of 5,200 nurses is anticipated.

HNHN will disseminate recruitment information via email regarding the opportunity to participate in the study. If the minimal sample size is not obtained via HNHN recruitment efforts, additional professional nursing organizations and health systems will be accessed, and the study team will use convenience sampling with the snowball method or social media advertising (if necessary). HNHN recruitment emails, using a random sample, offer a direct link to the consent form (S3 File). For participants in the convenience sample (non-HNHN recruitment source), a delayed recruitment method will be utilized to manage the flow of participants. The delayed recruitment will collect email addresses of potential participants in REDCap. Nurses will receive an acknowledgement of providing their contact information by email. As available and appropriate, email invitations to consent form will be sent to potential participants via email. If study is no longer accepting participants, nurses will be notified via email and provided with mental health resources.

Nurses who consent to participate in the study will be screened for participation through the mISP. Eligible participants who provide consent will be called by a study team member to ensure that any questions about the study are answered. Inclusion criteria for participation is currently working as a nurse with a license to practice and being identified by the mISP as having moderate to high risk of suicide (i.e., mISP designated Tier 1 or Tier 2). Nurses identified as having minimal to no distress (i.e., mISP designated Tier 3) are excluded, as are nurses who have previously participated in the MINDBODYSTRONG program. Table 1 describes how the mISP determines tier designation and level of distress.

**Table 1:** Determining mISP tier designation and level of distress.

	Level of Distress
**Tier 1**	
**1A**	◼ High distress with current suicidal ideation, plans, or behaviors.
	◼ PHQ-9 score ≥ 15 with intense feelings of anger, panic, anxiety, desperation, loss of control, or hopelessness.
**1B**	◼ High distress that does not include suicidal ideation, plans, or behaviors.
	Or
	◼ Prior suicide attempts and PHQ-9 score of 10–14.
	◼ Intense feelings of anger, panic, anxiety, desperation, loss of control, or hopelessness.
	Or
	◼ The current problems make it challenging to function.
**Tier 2**	◼ No current suicidal ideation, plans, behaviors, or prior attempts and moderate distress.
	◼ PHQ-9 score of 10–14.
	Or
	◼ Issues relating to alcohol or drug use or eating.
	Or
	◼ The current problems make is somewhat difficult to function.
**Tier 3**	◼ Minimal to no distress

Due to ethical considerations by the researchers, a therapist will contact all nurses who complete the mISP screening, no matter tier designation, as completing the mISP indicates that the individual may have some level of concern about their mental health. Participants meeting the inclusion criteria will continue the study while those meeting the exclusion criteria will be thanked for their time and provided with applicable resources outside of the study as determined by the therapist.

The study will operate out of The Ohio State University. Participants are currently being recruited and enrolled; however, recruitment and data collection have not been completed, nor have results been generated. The first participant was enrolled on October 26, 2023.

### Randomization

A computerized randomization scheme using permuted block randomization with varying block sizes of 2 or 4 is being implemented. Nurses in each block are randomly allocated 1:1 to the MINDBODYSTRONG^©^ and mISP program or mISP alone. Randomization is not controlled by any of the variables of interest in the study.

To ensure allocation concealment, the study team’s statistician developed the randomization scheme using SAS statistical software and input the coding into the randomization module in REDCap. The statistician is not involved with recruitment nor determining participant eligibility. The remaining study team members, in addition to the participants, are not privy to the randomization scheme. Awareness to group allocation only occurs after randomization.

To avoid techniques from the MINDBODYSTRONG© program being used with the attentional control group, the therapist is blinded to the approach used in the MINDBODYSTRONG© program. The therapist is also blinded to participant group allocation.

### Instruments and measures

The measures are administered electronically by study personnel via the mISP and REDCap. The mISP is used to determine participant tier designation, study eligibility, and to facilitate anonymous messaging with the therapist. Because data cannot be linked over time in the mISP, the measures are collected again in REDCap at baseline, immediately after program completion at week 8, and again at months three, six, and 12. The objective of the study measures is to evaluate any changes in mental health over time and assess **participants’ experiences with the interventions.**

#### Demographics

Demographic information collected will include gender, age, position, zip code, current mental health treatment, years as a nurse, and years practicing direct patient care.

#### mISP questionnaire

The mISP will identify nurses with moderate to high-risk suicide [36] via an objective, on-line screening questionnaire. Upon completion of screening, the mISP immediately outputs a tier designation based on participant responses that is only viewable to the therapist. The questionnaire will assess for intense emotional distress such as depression, anxiety, panic, rage, hopelessness, desperation, and loss of control which have been linked to depression with suicidal ideation. The participants will also respond to questions indicating burnout, substance use, disordered eating behaviors, post-traumatic stress disorder, current suicidal thoughts, behaviors, and plans, and past suicide attempts. This questionnaire is only taken once and is used to determine tier designation and study eligibility.

#### Patient health questionnaire (PHQ-9)

Depression will be assessed through the PHQ-9 [37] which is included in the mISP. This tool has been widely used in studies and has strong Cronbach’s alpha is reported above .90. It is scored based on a 4-point Likert scale with results categorized into five groups. Responses of 0–4 are considered minimal depressive symptoms, 5–9 are considered mild depressive symptoms, 10–14 are considered moderate depressive symptoms, 15–19 are considered moderately severe depressive symptoms, and greater than 20 are considered severe depression symptoms.

#### Columbia suicide severity rating scale

Suicidal intent will be measured with the valid and reliable Columbia Suicide Severity Rating Scale [38]. This is a semi-structured list of yes or no questions intended to assess the severity of suicidal ideation and suicidal behaviors. Questions are divided into four constructs: suicidal ideation, intensity of suicidal ideation, suicidal behavior, and lethality. The suicidal lethality construct uses a subscale rather than yes or no questions to track actual lethality.

#### Generalized anxiety disorder scale (GAD-7)

The valid and reliable GAD-7 [39] will be used to assess anxiety. This tool uses a 4-point Likert scale to measure feelings and actions associated with anxiety in the prior two-week period. The GAD-7 is widely used in research and in the field of mental health with Cronbach’s alpha is typically reported above .85.

#### Single-item burnout question

The following non-proprietary, single-item burnout question will be used: “Overall, based on your definition of burnout, how would you rate your level of burnout?” In a study of 5,404 participants, including 1,769 providers and 1,380 registered nurses, Dolan and colleagues [40] found that the single-item measure had a correlation of 0.79, sensitivity of 83.2%, specificity of 87.4%, and AUC of 0.93 (p  =  0.004) when compared to proprietary Maslach Burnout Inventory Scale.

#### Primary care PTSD scan for DSM-5 (PC-PTSD-5)

The PC-PTSD-5 is a 5-item screening tool to assess for probable PTSD in a primary care setting [41]. The instrument first asks about exposure to traumatic event to establish criteria for PTSD before asking five yes or no questions regarding PTSD symptomology. Recent studies have found the scale helpful in screening nursing and medical staff exposed to COVID-19 [42–45].

#### Job satisfaction scale (JSS)

The JSS is a 7-item scale using a 5-point Likert scale to determine the extent of satisfaction one feels towards their job [46]. A higher score indicates a higher level of job satisfaction. Cronbach alphas are typically reported as .80 and above.

#### Personal beliefs scale

The 10-item Personal Beliefs Scale was derived from the valid and reliable 16-item Healthy Lifestyle Beliefs Scale [47] and taps cognitive beliefs about dealing with stress and handling life’s problems (e.g., “I know how to deal with things that bother me in a healthy way”; “I am sure I can handle my problems well”). The Personal Beliefs Scale uses a 5-point Likert scale ranging from 1 (*strongly disagree*) to 5 (*strongly agree*). Eight specialists established content validity of the scale, and Cronbach’s alpha has consistently exceeded 0.80.

#### Healthy lifestyle behaviors scale

The Healthy Lifestyle Behaviors scale is a 16-item measure that assess the degree of engagement with healthy lifestyle behaviors (e.g., making healthy food choices and exercising). It is scored on a 5-point Likert scale ranging from 1 (*strongly disagree*) to 5 (*strongly agree*) [48]. The Cronbach alphas are reported at .80 and above.

#### Modified social readjustment rating scale

The Social Readjustment Rating Scale is a 43-item scale that identifies the frequency of various life events over the prior 12 months. The scale is intended to provide a quantitative view of life events that required the subject to make psychological adjustments that have been associated with the timing of illness development [49]. This scale has been modified to 10 life events for this study to reduce participant burden.

#### Open-ended stressors question

There will be one previously tested open-ended, self-report question regarding stressors [25, 50, 51]. The question is “Please take a minute to let us know about anything that has been particularly stressful for you lately–death of a loved one, relationship break-up, academic stressors, family or money problems, difficulty with your living situation–or anything else that might be contributing to how you are feeling”.

#### Program evaluation questionnaires

Feedback will be gathered regarding the participants’ experiences with the interventions in terms of their helpfulness (S4, S5 Files). These questionnaires will also gather data on any additional treatment, such as therapy or medication, the nurses may have received since the beginning of the study.

#### MINDBODYSTRONG^©^ facilitator responses

Qualitative data will be collected from participants during scheduled facilitator sessions regarding the participants experience with MINDBODYSTRONG^©^ and their progression through the program (S6 File).

### Processes and interventions

#### mISP screening and referral program

Once the questionnaire is submitted by a participant, it is automatically scored and stratified into one of four tiers by the mISP program: 1A, 1B, 2, and 3. Tier 1A and 1B are both participants in high distress, or high risk, differentiated by any level of current suicidal ideation. Tier 2 are participants at moderate risk of suicidal ideation without a history of suicide attempt or any current suicidal ideation. Respondents who do not meet any of these criteria are considered low risk and designated tier 3.

The therapist is notified immediately when a questionnaire is available on the mISP platform. The notification tells the counselor the tier of the participant and includes access to the participant’s responses to the questionnaire. The therapist will engage with each participant with a detailed assessment and personalized response after reviewing the participant’s questionnaire. Tier 1A and 1B participants are responded to within 24 hours, tier 2 within 36 hours and tier 3 within 48 hours.

Participants can review the therapist’s response by logging back into the mISP platform. All participants, regardless of tier, are invited to continue interacting with the therapist. Additional responses from the participants are answered within 24 hours. If identified as having moderate to high risk of suicide, tier 1A or 1B, participants can further engage with the counselor through the mISP or over the phone for support and assistance finding mental health resources. When indicated, the therapist will direct the participant to contact 988, the crisis hotline number, and encourage the participant to use their insurance benefits and/or employee assistance program to obtain local treatment. The therapist will provide referrals and bridge high-risk participants into treatment.

#### MINDBODYSTRONG^©^

Digitized MINDBODYSTRONG^©^ is hosted on an Ohio State University eLearning website. Participants must create an account to access the learning materials. There is no limit on the number of times participants can access the learning materials. Participants are encouraged to complete one learning module per week and completion reminders are sent weekly via email with or without text (based on participant preference) for eight weeks. Learning modules are video recordings varying from 9 to 20 minutes in length. Table 2 provides further details concerning the weekly modules.

**Table 2:** MINDBODYSTRONG^©^ learning module topics by week.

	Topic (length of module in minutes)
**Week 1**	◼ Thinking, Feeling, and Behaving (20 mins)
**Week 2**	◼ Self-Esteem and Positive Thinking/Self-Talk (11 min)
**Week 3**	◼ Stress and Coping (18 min)
**Week 4**	◼ Problem Solving & Setting Goals (10 min)
**Week 5**	◼ Dealing with you Emotions in Healthy Ways through Positive Thinking and Effective Communication (15 min)
**Week 6**	◼ Coping with Stressful Situations and Valuable Sleep (9 min)
**Week 7**	◼ Pulling it All Together for a Healthy YOU! (9 min)
**Week 8 (optional)**	◼ Post-Traumatic Stress Disorder (16 min)

MINDBODYSTRONG^©^ is completed asynchronously by the participant over eight weeks. A trained MINDBODYSTRONG^©^ facilitator checks in with participants over the telephone at baseline at weeks three and five of the online program to reinforce key program concepts and assess whether participants are completing the weekly skills building activities. Participants are informed that total time commitment per week is about 45 minutes, including time to complete learning modules, skills building activities, and facilitator discussions.

### Data management

Collected data will be encrypted and stored on a secure server which is located at the institution of the first author. Only the PI, external collaborators, and key personnel will have access to the electronic data and de-identified data. Data shared between study sites will be done via a password protected computer and server.

No datasets were generated or analysed during the current study. Because data deals with sensitive mental health information, data will not be made publicly available to protect participant identities. Data requests can be made by contacting the primary author. The final dataset will be de-identified and coded using unique identifiers to preserve participant confidentiality prior to release for sharing. Even with de-identification, the possibility of deducing participants with unusual characteristics remains. Thus, the data and associated documentation will only be made available to users under a data sharing agreement that provides for: (1) a commitment to using the data only for research purposes and not to identify any individual participant; (2) a commitment to securing the data using appropriate computer technology; and (3) a commitment to destroying or returning the data after analyses are completed. Given all data will be collected online no hard copies of the data will be obtained.

### Data analysis

We will conduct intent-to-treat analyses. Descriptive statistics will be first used to examine variable distribution, identify any data abnormality such as outliers, and summarize sample characteristics, stratified by intervention and control groups. Appropriate data transformation will be performed if needed to achieve normality. For each outcome variable, mixed-effects linear regression modeling will be used to fit the outcome as a linear function of intervention, time, intervention dose, and their interactions. Generalized linear mixed-effects regression with appropriate link function will be used if data fail the normality assumption. From the model, we will obtain estimates on the between-group difference in the change of outcome measure from baseline at each follow-up time point and estimates on the dose effect of the completed sessions in the MINDBODYSTRONG^©^ program. The model will be further extended to adjust for covariates, examine nonlinear relationships, and explore the heterogeneity of intervention effects across subgroups.

The study has seven outcomes and four between-group comparisons of pre-post changes for each outcome. Therefore, multiple testing adjustment to keep the study-wide type I error rate under 0.05 will be employed. Missing data due to attrition at longitudinal follow-up is expected. We will examine the extent and pattern of missing data, and perform appropriate multiple imputation according to missing data mechanism. The mixed-effects regression modeling allows for missing at random. This analysis will be repeated with and without multiple imputation. If missing not at random exists, pattern mixture modeling will be used instead. Sensitivity analysis will be used to examine the robustness of study findings before vs. after multiple imputation or under pattern-mixture modeling. We will use SAS 9.4 for all quantitative analysis. We will conduct two-sided tests and use Bonferroni adjusted p-values < 0.007 for statistical significance to keep the study-vide type I error rate under 0.05. Due to the conservative nature of Bonferroni adjustment for multiple testing, we will conduct sensitivity analysis using other approaches (e.g., Hommel method) [52]. Regardless of statistical significance, we will report point estimates, their precision (e.g., 95% confidence intervals), and effect sizes.

Qualitative data from the open-ended stressors question will be analyzed through inductive reflexive thematic content analysis using a manual method (no software required) [53, 54].

### Safety and risk considerations

Participants may acquire valuable information about their personal health and wellness that they may use in their future by consenting to the study. The surveys, screeners, and assessments selected for this study pose minimal risk to participants. However, the surveys, screeners, and assessments may result in uncomfortable emotions for the participant. Thus, a Data and Safety Monitoring Plan and Board have been established for this study to protect the health and safety of participants and provide information relevant to their participation. Participants will also be provided with a set of mental health care resources.

At the time of consent, participants are made aware that the mISP program and the corresponding study are not crisis response services, and that immediate availability of the therapist should not be expected. Participants identified as having moderate to high risk of suicide will be contacted via the encrypted mISP program within 24 hours during the work week, however there is no guarantee that the therapist will be available to address any safety concerns before then, including imminent suicide risk or other mental health crisis. The therapist, facilitation, and investigators will make best efforts to assist the participants with tools, resources, and referrals to protect themselves in crisis situations, but it is dependent on the participants to interact with study personnel and follow through to keep themselves safe.

The information shared by the participants and the data compiled is collected and stored online; therefore, there is a potential risk of data breach. AFSP has created an individualized version of the mISP specific to this study to assist in protecting the data acquired within the mISP. AFSP will maintain the encryption of the mISP program so that communications between the participant and therapist are protected, and the identity of the participant is kept private.

### Ethical considerations

This proposed study was reviewed and approved by The Ohio State University Institutional Review Board, Protocol Record 2021B0417.

## Discussion

The mISP plus MINDBODYSTRONG^©^ RCT will examine the efficacy of a digitized, cognitive-behavioral skill building program combined with the encrypted, de-identified screening and referral program on depression, suicidal ideation, burnout, anxiety, personal cognitive beliefs, healthy lifestyle behaviors, and job satisfaction in nurses. These are pressing issues as mental health concerns, including suicidal ideation, depression, anxiety, and burnout, among nurses are currently a public health epidemic.

Mental health concerns, including suicidal ideation, ebb and flow through the lifespan. Research shows that a prior history of suicide attempts exponentially raises the risk of future suicide attempts [55]. The use of a cognitive-behavioral skill building program is impactful because the skills that participants learn and practice can be used again with future distress. Paired with the de-identified screening and referral program, those who may not have reached out for help will have timely access to assistance that can benefit them.

If found to be effective, this combination of interventions can be scaled to assist nurses through employers and providers across the U.S. A recent meta-analysis showed that the issues with high rates of depression, anxiety burnout, and suicidal ideation in nurses are not unique to the United States but seen internationally [56]. With appropriate translation and trials, there is potential for this unique combination to have a positive effect on the lives of nurses across the globe.

Findings from this study will primarily be disseminated through publications and presentations. Any needed amendments to the study, including termination, will be handled via The Ohio State University’s Institutional Review Board.

### Limitations

RCTs are the gold standard of intervention evaluations, however, this proposed protocol has limitations. While randomization at the national level is being utilized, the sample may not be fully representative of the American nurse population. As the study focuses on individuals with moderate to high risk of suicidal ideation, the findings of the intervention cannot be generalized to those at low risk of suicidal ideation. Study findings could be biased as those completing the intervention may be more motivated to obtain help and work on their mental health. Not all individuals with mental health conditions or suicidal thoughts are ready or capable to engage in a 7-week program intervention with the option for an additional module. mISP and MINDBODYSTRONG^©^ are both accessed digitally, which may leave out potential study participants who do not have the proper technological skills or equipment (computer or smart phone). Participating in remote activities like those described in this protocol may not appeal to nurses who spent the last few years socially distancing themselves due to COVID-19 restrictions. These nurses may crave in-person interactions and find digital communications fatiguing. The touchpoints selected for post intervention follow-up (8 weeks, 3,6, and 12 months) may also be a limitation. It is possible that the study team may not identify effects over time with the longer check-in points. Therefore, it is suggested that researchers interested in using this protocol utilize shorter check-in points (e.g., 15, 30, 60, 90, 120, 180, 200…days). Lastly, attrition may also occur, which could limit the strength of the study findings. However, incentives and multiple check-in points are being used to reduce attrition.

## Supporting information

S1 File SPIRIT 2013 checklist: Recommended items to address in a clinical trial protocol and related documents*. *It is strongly recommended that this checklist be read in conjunction with the SPIRIT 2013 Explanation & Elaboration for important clarification on the items. Amendments to the protocol should be tracked and dated. The SPIRIT checklist is copyrighted by the SPIRIT Group under the Creative Commons “Attribution-Non Commercial-NoDerivs 3.0 Unported” license.
(DOC)

S2 File IRB approved protocol. (DOCX)

S3 File The Ohio State University consent to participate in research. (DOC)

S4 File MINDBODYSTRONG^©^ program evaluation questionnaire. (DOCX)

S5 File Modified interactive screening program evaluation questionnaire. (DOCX)

S6 File MINDBODYSTRONG^©^ facilitator responses. (DOCX)
