# Supplementary material for: A study protocol for the modified interactive screening program plus MINDBODYSTRONG© RCT: A mental health resiliency intervention for nurses
Source: PLoS One. 2024 Jun 6;19(6):e0303425. doi: 10.1371/journal.pone.0303425 (PMC11156330; doi:10.1371/journal.pone.0303425)
Supplement: S2 File — (DOCX) [file pone.0303425.s002.docx]

**S2 IRB Protocol**

**Study Title:** Modified Interactive Screening Program plus MINDBODYSTRONG: A Mental Health Resiliency Intervention for Nurses

**I. Background**

Suicide is the 10^th^ leading cause of death in the U.S., with nurses having a higher suicide rate than the general population (Davidson, 2020). Nurses (the largest healthcare workforce in the country with over four million) experience high levels of stress, burnout, and depression, which have escalated since the COVID-19 pandemic started (Kirzinger et al., 2021; Melnyk et al., 2021a; Pappa et al., 2020). Recent studies by our team and others have reported the devastating toll that the pandemic has had on the profession of nursing that is resulting in nurses deciding to leave the profession (Melnyk et al., 2021a; Preti et al., 2020; Turale & Nantsupawat, 2021; U.S. Department of Health and Human Services [HSS] Office of Inspector General, 2021). Findings from our recent study of nurses on the COVID-19 front line revealed that the pandemic negatively impacted their mental and physical health with 65% suffering from burnout (Melnyk et al., 2021a).

The modified American Foundation of Suicide Prevention (AFSP) Interactive Screening Program (mISP) program is a tested method of screening to detect clinicians at moderate to high risk for suicide and referring them for treatment through an encrypted de-identified on-line platform (Davidson et al., 2018a; Davidson, Accardi, Sanchez, Zisook, & Hoffman, 2020; Downs et al., 2014; Martinez et al., 2016; Moutier et al., 2012; Norcross et al., 2018; Pospos et al., 2019; Zisook et al., 2015).

MINDBODYSTRONG^©^ is an adaptation of a well-tested cognitive-behavioral skills building intervention (also known as COPE in the literature) that provides a cognitive-behavior theory-based approach to decrease depression, anxiety and suicidal ideation and improve healthy lifestyle beliefs and behaviors in at-risk populations. It consists of seven manualized sessions that can be delivered by health professionals other than psychiatric mental health providers, making it more feasible for wide-scale implementation to nurses in healthcare systems across the U.S. Findings from a recent randomized controlled trial (RCT) with newly licensed registered nurses demonstrated that group delivery of MINDBODYSTRONG^©^ significantly decreased depression and anxiety as well as improved healthy lifestyle beliefs, behaviors and job satisfaction at three and six months following completion of the intervention (Sampson et al., 2019; Sampson et al., 2020). However, it is not known whether an on-line version of MINDBODYSTRONG^©^ combined with mISP screening can produce the same outcomes in clinicians self-reporting moderate to high symptoms of depression.

***Purpose***

The proposed study is innovative in that it will be the first to test the combination of the mISP program with a new on-line digitalized version of the previously tested manualized version of the CBSB program (i.e., MINDBODYSTRONG^©^) for nurses. If found to be effective, the combined program will be able to be easily scaled to prevent suicide in nurses and potentially other high-risk populations of clinicians. Although CBT is gold standard evidence-based treatment for depression, only a small percentage of affected individuals receive it due to a shortage of mental health providers, which contributes to high depression relapse rates of 50 to 70%. A scalable digitalized intervention such as that which is being proposed could be one key solution to bringing CBT skills to nurses at risk for suicide who could so benefit from them.

***research aims***

The specific aims of this study are to:

1. Assess acceptability and usability of a digitalized on-line interactive version of the MINDBODYSTRONG^©^ cognitive-behavioral skills building program with at least 10 nurses, and
2. conduct a rigorous randomized controlled trial (RCT) to determine the effects of the modified American Foundation for Suicide Prevention’s (AFSP) Interactive Screening program (mISP) combined with the on-line version of the MINDBODYSTRONG^©^ program versus the mISP program alone on depression, suicidal ideation, burnout, anxiety, post-traumatic stress, healthy lifestyle beliefs, healthy lifestyle behaviors, and job satisfaction in U.S. nurses.

**II. Methodology**

***Design***

*aim 1*

In order to achieve aim 1, the newly digitalized version of MINDBODYSTRONG^©^ will be evaluated by at least 10 nurses for acceptability and usability. Feedback will be used to refine the program.

*aim 2*

For aim 2, the full-scale study will use a two-arm RCT design in which nurses who are identified as having moderate to high risk of suicide, as identified by the mISP, will be randomly allocated to the MINDBODYSTRONG^©^ program or the attention control group (the mISP program alone) with post-intervention follow-up at 8 weeks, three, six and 12 months.

***Sample and setting***

*aim 1*

At least ten volunteer nurses will test the usability and acceptability of the newly created digital version of MINDBODYSTRONG^©^.

*aim 2*

The sample for the RCT will be comprised of nurses recruited nationally from nursing professional organizations such as Healthy Nurse Healthy Nation, a program of the American Nurses Association Enterprise, a national professional organization that represents the interest of the nation’s four million registered nurses, health systems where they are employed, or social media if needed. Nurses must be 18 years or older, have not participated in either the MINDBODYSTRONG program or this study previously.

A minimal sample of 364 nurses (182 per arm) will be needed to obtain a sample that completes at least six of the seven MINDBODYSTRONG^©^ sessions. To achieve a sample size of 364 nurses, we anticipate needing to sample a random sample of a total of 15,600 nurses. In addition to the random sample, we will recruit a convenience sample outside of HNHN using a snowball method of recruitment from nursing professional organizations, health systems, and social media if needed.

***Recruitment, data collection and the intervention***

*aim 1*

Nurses in aim 1 will be recruited via electronic newsletters, flyers or recruitment verbiage on various OSUMC web-platforms or distributed by OSUWMC personnel, such as department Buckeye Wellness Innovators or Magnet representatives, through the nurse residency program, and shared governance or research councils. Communications will include information about the opportunity to participate in the study and a link to consent to the study. Nurses who are interested in participating in the study will be asked to click on a link included on these communication platforms and provide their name, contact information and basic demographic information. Upon completion of this form consenting nurses will receive further information about participating in the digitized MINDBODYSTRONG intervention.

Digitalized MINDBODYSTRONG™ consist of 8 sessions, 7 sessions of MINDBODYSTRONG plus an additional session addressing coping with trauma. Participants will complete one session, weekly. Nurses will matriculate through sessions 1-8 via an online platform. Reminders will be sent weekly to complete the next MINDBODYSTRONG© session and a MINDBODYSTRONG© facilitator will check in with participants by phone at baseline, weeks 3 and 5 of the on-line program to reinforce key program concepts and assess whether participants are completing the weekly skills building activities.

At the end of the MINDBODYSTRONG™ program, participants will be sent an email asking them to complete an evaluation of the digitized version of the MINDBODYSTRONG™ program. All data will be collected via REDCap.

*aim 2*

An opportunity to participate in this study will be shared by a professional nursing organization, such as HNHN, or health system to its members via email. Social media advertising will utilized if necessary. HNHN recruitment emails in random sample offer direct link to consent form. For participants in the convenience sample (all other recruitment sources), a "delayed recruitment" method will be utilized. This method will collect email addresses of potential participants in REDCap. Nurses will receive an acknowledgement of providing their contact information by email. As available and appropriate, email invitations to consent form will be sent to potential participants via email. If study is no longer accepting participants, nurses will be notified via email and provided with mental health resources. Nurses who consent to participate in this study will be screened with the modified interactive screening program (mISP). Participants will complete questions associated with the mISP. They will receive a telephone call to review study procedures given the multiplatform format of the study.

Participants who are identified as having moderate to high risk of suicide, as identified by suicide risk on the mISP, will be contacted by a mental health counselor who will engage them through the encrypted interface online. The counselor will offer support online or by phone. Where indicated, the mental health counselor will provide the crisis hotline, and encourage participants to use their insurance provider and/or Employee Assistance Program to obtain treatment. The mental health counselor will offer to help with referrals and will bridge high-risk participants into treatment. Participants who are **not** identified as having moderate to severe risk of suicide will be thanked for their time and provided non-study resources by mISP counselors.

Participants, identified as moderate- to high-risk, will also be asked to complete additional surveys via validated instruments which assess depression, suicidal ideation, burnout, anxiety, healthy lifestyle beliefs, healthy lifestyle behaviors and job satisfaction. Some questions asked in the mISP will be repeated in the baseline surveys provided to patients to allow for data analysis. The anonymous nature of the mISP prevents comparison of these responses longitudinally at the participant level. Further, individuals identified as moderate-to high- risk of suicide will be randomly assigned to either the intervention or control groups following the mISP screening. Participants randomized to the MINDBODYSTRONG^©^ program (intervention) will receive the 7-session online interactive program with an option for an 8^th^ module that focuses on how to deal with post-traumatic stress symptoms. Reminders will be sent weekly to complete the next MINDBODYSTRONG^©^ session and a MINDBODYSTRONG^©^ facilitator will check in with participants by phone at baseline, weeks 3 and 5 of the on-line program to reinforce key program concepts and assess whether participants are completing the weekly skills building activities. Nurses assigned to the control group will only receive the mISP program alone (aforementioned) and will be contacted for follow-up surveys.

Baseline data will be collected via an on-line confidential survey among all participants, immediately after participants consent to participate in the study. The survey will be administered at baseline (T0) 8 weeks (T1), three months (T2), six months (T3) and 12 months (T4). After completing surveys, participants will be emailed mental health resources for their use. Participants with elevated depression or anxiety screenings will receive an email including notification of their elevated screening result and a link to return to the mISP platform to connect with a study counselor. All participants will receive study messages at five months and nine months of participation to prevent study attrition.

***Instrumentation and measures***

Baseline assessment will include demographics and mental health outcomes using the mISP questions. Limited demographics will be collected intentionally to promote survey completion given the risk of alienating those who might be concerned about disclosing mental health issues. Demographics will include zip code, years as a nurse, and years practicing direct patient care.

**Suicide Risk (The ISP Questionnaire).** Suicide risk categories will be provided by the **m**ISP platform (Mortali & Moutier, 2018), which includes an on-line screening questionnaire, initially designed and developed by the American Foundation for Suicide Prevention (AFSP). The questionnaire contains the 9-item Patient Health Questionnaire (PHQ-9); measures of intense emotional distress (anxiety, panic, rage, hopelessness, desperation and loss of control) that have been linked to depression with suicidal ideation (SI); burnout; alcohol and drug use; disordered eating behaviors; post-traumatic stress disorder; current suicidal thoughts, behaviors, and plans and past suicide attempts; current mental health treatment; and demographic items, including gender, position (fellow, faculty, nurse,) and age.

Once participants submit their questionnaire, it is automatically analyzed and stratified into one of four tiers of distress: Tier 1A, Tier 1B (both high distress), Tier 2 (moderate distress), and Tier 3 (low distress). Criteria for Tier 1 (high risk) include a PHQ-9 score of 15 or higher, current SI, a PHQ-9 score of 10–14 with prior suicide attempt, intense feelings of anxiety, panic, rage, desperation, or loss of control, or an indication that current problems make it very or extremely difficult to function. Tier 1 is further divided into Tier 1a, which indicates any level of current SI, and 1b, which indicates suicide risk and severe distress without current ideation. Criteria for Tier 2 (moderate risk) include a PHQ-9 score of 10–14 without a history of suicide attempt or current SI, problems related to alcohol or drug use or eating behaviors, or an indication that current problems were making it somewhat difficult to function. Respondents who do not meet any of these criteria are designated as Tier 3 (low risk).

Immediately after the questionnaire is posted to the mISP platform, the computer system generates notifications to the designated program counselors. The email notifications indicate each participant’s tier level and provide a link to the participant’s record on the mISP platform. Program guidelines call for all Tier 1 participants to be answered within 24 hours, Tier 2 participants within 36 hours and Tier 3 participants within 48 hours. Counselors review the participants’ questionnaires and create a detailed, personalized response and assessment for each participant, using a template specific to the participant’s distress tier, which encourages interaction between participant and counselor. In addition to the assessment, the counselor addresses any questions or comments left by participants in an open-ended comment box at the end of the questionnaire. Participants are invited to exchange dialogue messages with the counselor using the mISP platform’s messaging system, or to contact the counselor directly using contact information provided by the counselor, including the counselor’s name, office location and phone number. Therefore, participants maintain the ability to keep their identity private.

The counselors’ responses are accessible to participants by logging back onto the program platform with their user ID and password. Participants who provide an email address automatically receive an email notification alerting them of the response with a link to the program platform. Participants can return independently to the platform and log in to view the counselor’s response, regardless of having provided an email address. All Tier 1 and Tier 2 participants are urged to contact the counselor to arrange an in-person or virtual meeting. All participants, regardless of tier, are offered the option of using the platform’s “dialogue” feature to communicate online with the counselor while keeping their identity private. In general, the counselor’s key aims in the responses are to convey interest, support and availability, and to encourage help-seeking, whether in-person or through the anonymous online dialogue.

**Depression.** The 9-item valid and reliable Personal Health Questionnaire-9 will be used to measure depressive symptoms. Participants are asked to respond to nine items (e.g., little interest or pleasure in doing things; feeling down, depressed or hopeless) regarding how they have felt over the previous 2-week period (Kroenke et al., 2003). Scores are based on a 4-point Likert scale ranging from 0 “not at all” to 3 “nearly every day.” Scores between 0-4 are considered minimal depressive symptoms, scores between 5-9 are considered mild depressive symptoms, scores between 10-14 are considered moderate depressive symptoms, scores between 15-19 are considered moderately severe depressive symptoms, and scores ≥20 are considered severe depressive symptoms. This tool has been used in multiple studies and has strong Cronbach alphas reported above .90.

**Suicidal Intent.** The valid and reliable Columbia Suicide Severity Rating Scale (Posner et al., 2011) assesses the severity of suicidal ideation and behavior through a semi-structured list of *yes* or *no* questions divided into four constructs: (1) suicidal ideation; (2) intensity of ideation; (3) suicidal behavior; and (4) suicidal lethality subscale (actual or potential). Example questions from constructs 1-3 include, *have you thought about being dead or what it would be like to be dead?* *How many times have you had these thoughts?* *Did you do anything to try to kill yourself?* *Has there been a time when you tried to end your life, but someone stopped you?* Construct 4, suicidal lethality, does not use yes or no questions, but rather it is a subscale used to track actual lethality (0 = no physical damage or very minor damage; 2 = moderate physical damage; 3 = moderately severe physical damage; 4 = severe physical damage; and 5 = death). If actual lethality is equal to 0, then potential lethality can be assessed (0 = behavior not likely to result in injury; 1 = behavior likely to result in injury, but not death; 2 = behavior likely to result in death despite available medical care). In the landmark Posner et al. (2011) multi-site study, the C-SSR demonstrated good convergent and divergent validity with other suicidal ideation and behavior scales, high sensitivity and specificity for suicidal behavior classifications when compared to another behavior scale and an independent suicide evaluation board, sensitivity to change over time, and strong internal consistency for the intensity of ideation subscale. Posner et al. (2011) did not examine interrater reliability due to study design restrictions, however, it has been demonstrated in other studies (Brent, 2009; Stavarski et al., 2011; Mundt et al., 2010).

**Anxiety.** The valid and reliable Generalized Anxiety Disorder Scale (GAD-7) (Löwe et al., 2008) will be used to measure anxiety. The GAD-7 measures feelings and actions associated with anxiety within the prior 2-week period. Responses are based on a 4-point Likert scale ranging from 0 (not at all) to 3 (nearly everyday). Scores between 5-9 were considerd mild anxiety, scores between 10-14 were considered moderate anxiety and scores ≥15 were considered severe anxiety. The GAD-7 is a widely used tool with Cronbach alphas typically reported above .85.

**Burnout.** The following non-proprietary single-item question from the Maslach Burnout Inventory will be used to measure burnout. In a study of 5,404 participants, including 1,769 providers and 1,380 registered nurses, the single-item measure had a correlation of 0.79, sensitivity of 83.2 %, specificity of 87.4 %, and AUC of 0.93 (p = 0.004) when compared to the full scale. Results were similar when stratified by respondent occupation (Dolan et al., 2015). **Overall, based on your definition of burnout, how would you rate your level of burnout?”** Responses are scored on a five-category ordinal scale, where 1 = “I enjoy my work. I have no symptoms of burnout;” *2 =* “Occasionally I am under stress, and I don’t always have as much energy as I once did, but I don’t feel burned out;” *3 =* “I am definitely burning out and have one or more symptoms of burnout, such as physical and emotional exhaustion;” *4 = “*The symptoms of burnout that I’m experiencing won’t go away. I think about frustration at work a lot;” *and 5 =* “I feel completely burned out and often wonder if I can go on. I am at the point where I may need some changes or may need to seek some sort of help.”

**Post-traumatic stress.** The Primary Care PTSD Screen for DSM-5 (PC-PTSD-5) will be used to screening for post-trauma symptoms. This 5-item screening tool was developed by Prins et al. (2015) to identify individuals with probable PTSD in a primary care setting. The measure asks a first question about whether an individual has had any exposure to traumatic events (giving examples). If the individual denies exposure, they score a zero. If they affirm some traumatic event, they answer yes or no to 5 items that are symptoms of post-trauma response over the past month; each yes is scored as one point. Preliminary results from validation studies indicate a cut-point of 3 indicates probably PTSD, while use of a cut-off of 4 is optimally efficient (Prins et al). Other recent studies have found the scale acceptable for screening in US veterans (Bovin et al., 2020); and nursing and medical staff exposed to Covid-19 (Amsalem et al., 2021; Huang et al., 2021; Mosheva et al., 2021; Vance et al., 2021).

**Job Satisfaction.** Job Satisfaction will be measured using the Job Satisfaction Scale (JSS) (Price & Mueller, 1983). This 7-item scale queries responses such as “I find real enjoyment in my job” and are measured on a 5-point Likert scale ranging from 1 (strongly disagree) to 5 (strongly agree). Cronbach alphas are typically reported as .80 and above.

**Personal Beliefs Scale.** Developed by the PI (Bernadette Melnyk), this 10-item scale will be used to tap cognitive beliefs about dealing with stress/problems and engaging in healthy lifestyle behaviors, the proposed mediator in this study. Participants respond to each of the items on a 10-point Likert scale ranging from (1) strongly disagree to (5) strongly agree (e.g., “I am sure I can handle my problems well.”; I know how to deal with things that bother me in a healthy way.” Content validity has been established with Cronbach alphas reported as .80 and above.

**Healthy Lifestyle Behaviors Scale.** The Healthy Lifestyle Behaviors scale was developed by Melnyk and colleagues (Melnyk et al., 2013a). The 16-item scale asks for responses on statements such as “I make healthy food choices” and “I set goals I can accomplish.” It us scored on a 5-point Likert scale ranging from 1 (strongly disagree) to 5 (strongly agree). The Cronbach alphas are typically reported as .80 and above.

**Life Stressors.** The Social Readjustment Rating Scale was developed to provide a quantitative assessment of life events requiring psychological adjustment that have been associated with the timing of development of illness (Holmes et al., 1967). It is a 43-item scale that records the frequency of occurance of various life events over the past twelve months. Each event is provided a different score based on its relative weight. To reduce participant burden, this scale has been modified to 10 life events.

**Open-ended stressors question.** One previously tested question on self-reported stressors (risk factors) will be used (Davidson et al., 2018; Liu et al., 2016; NAM, 2016). The question is: **Please take a minute to let us know about anything that has been particularly stressful for you lately – death of a loved one, relationship break-up, academic stressors, family or money problems, difficulty with your living situation – or anything else that might be contributing to how you are feeling.**

**Evaluation Questionnaire.** Questionnaires also will be administered to yield open ended feedback on the interventions in terms of their helpfulness as well as collect data on whether the nurses have received any treatment since beginning the study (e.g., therapy, medication).

**MINDBODYSTRONG Facilitator Responses.** Qualitative data from participants regarding their experience with the MINDBODYSTRONG and progression through the program will be collected during scheduled facilitator sessions.

***data management***

*aim 1*

All data will be collected via REDCap. Data will be stored on a secure password protected computer and server. Only the PI, external collaborators and key personnel will have access to the data. Project results may be used in reports, presentations, or publications and the data from this survey will be de-identified.

*aim 2*

The mISP will be used to collect data on the mISP specific questions. All other surveys will be administered via REDCap and presented to those who screen in the moderate to high group. Research data will be collected and linked at each time point for analysis.

The AFSP will manage the encryption of the mISP so that communication can occur between participants and the therapists in a manner where the participant can keep their identity private if desired. The format of the program is necessary for successful recruitment to overcome barriers associated with stigma against mental health treatment.

Data will be stored on a secure password protected computer and server. Only the PI, external collaborators and key personnel will have access to the data. Project results may be used in reports, presentations, or publications and the data from this survey will be de-identified.

**III. Analysis**

Congruent with the RCT study design, we will conduct an intent-to-treat analysis. Descriptive statistics will be first used to examine variable distribution, identify any data abnormality (e.g., outliers), and summarize sample characteristics, stratified by intervention and control groups. Appropriate data transformation will be performed if needed to achieve normality. Bivariate tests (two-sample t-test or Chi-square test) will be used to compare the balance of baseline measures between the intervention and control groups. For each outcome variable, we will use mixed-effects linear regression modeling to fit the outcome as a linear function of intervention, time (baseline, immediate post-intervention at 8-weeks, 3 months, 6 months, and 12 months), intervention dose, and their interactions. From the model, we will derive between contrast estimates on the intervention effect (that is, the between-group difference in the change of outcome measure from baseline) at each follow-up time point (immediate post-intervention, 3 months, 6 months, and 12 months) and estimates on the effect of dose (that is, number of completed sessions of iCBT program) on intervention effect (dose-response relationship). We will further extend the model to adjust for covariates, examine nonlinear relationship, and explore the heterogeneity of intervention effects across subgroups. Should any outcome be dichotomized, we will use mixed-effects logistic regression. The study has seven outcomes and four between-group comparisons of pre-post changes for each outcome. Therefore, we will employ multiple testing adjustment to keep the study-wide type I error rate under 0.05. We expect missing data due to attrition at longitudinal follow-up. We will carefully examine the extent and pattern of missing data and perform appropriate multiple imputation. The mixed-effects regression modeling allows for missing at random. We will repeat the analysis with and without multiple imputation. If missing not at random exists, we will use pattern mixture modeling instead. Sensitivity analysis will be used to examine the robustness of study findings before vs. after multiple imputation or under pattern-mixture modeling. Qualitative data from one open-ended question of stressors will be analyzed through thematic content analysis, and also sorted into work, home, and mixed work/home stressors. Given that it is now known that nurses experience job problems prior to suicide more than the general population, this data will also help to inform the nation of the nature and context of job issues that high risk nurses disclose.

**IV. Study Team and Roles**

The Ohio State University will operate the iterative cohorts of the MINDBODYSTRONG^©^ on-line cognitive-behavioral skills building seven-week program with accompanying session addressing trauma. Drs. Bernadette Melnyk, Jacqueline Hoying, Sharon Tucker, and Marlene Sampson will support data collection, analysis and report writing from the data collected during and following the skills-building intervention. The statistician for the grant (Dr. Alai Tan) will operate from The Ohio State University and analyze all sets of data. The project coordinator and mental health counselors will be housed at The Ohio State University.

Drs. Judy Davidson and Sidney Zisook of the University of California San Diego provide expertise from their experience operating the Healer Education Assessment and Referral screening, risk detection and referral program. Dr. Sidney Zisook will advise the mental health counselors who will communicate with screening participants. Dr. Judy Davidson will support data collection, report writing from the data collected during screening, and also co-liaison with the Healthy Nurse Healthy Nation and other nursing professional organizations, as needed, to coordinate the screening process. Dr. Zisook will support report writing.

**V. Risks and Benefits**

Participants may learn valuable information about their personal health and wellness. The surveys participants will be asked to complete pose minimal risk. Participants identified as having moderate to severe risk of suicide you will be contacted via the encrypted platform within 24 hours; however, it is not guaranteed that a counselor will be able to address any imminent suicide risk before then. The mISP provided by AFSP is not a crisis intervention service; therefore, no follow-up services will be provided unless requested. Given data is collected and stored online there is a potential risk of a data breach.

**VI. Data Safety Monitoring Plan**

Data will be stored on a secure password protected computer and server. Only the PI, external collaborators and key personnel will have access to the data. REDCap will be used to house data among participants enrolled in both aims of the study. An individualized ISP will be built specifically for this project by AFSP for data collected for aim 2.

Data shared between study sites will be done via a password protected computer and server. Copies of reports, presentations and publications (all with de-identified data) will be shared via email communication and the server. Given all data will be collected online no hard copies of the data will be obtained. Project results may be used in reports, presentations, or publications and the data from this survey will be de-identified.

A Data Safety Monitoring Plan and Board will be established for this study. The OSU College of Nursing has implemented a Data and Safety Monitoring Committee (DSMC) to protect the health and safety of human participants and provide information relevant to participants’ continuation in clinical studies. The progress of the study will be monitored by the college’s DSMC, comprised of Dr. Mary Beth Happ, Senior Advisor for Research Administration, Dr. Nathan Helsabeck, statistician and assistant research professor, and the director of the college’s psychiatric nurse practitioner program, Dr. Barbara Warren, who is a psychiatric mental health clinical nurse specialist as well as an additional member from University of California, San Diego, who is a Professor in the Department of Psychiatry, Dr. Colin Depp. On a quarterly basis, the Co-Principal Investigators (co-PIs), and project coordinator will prepare a written report on the progress of the study, including data on: enrollments, comparison of target to actual enrollment, overall status of the study participants, information on race/ethnicity and gender, information on school(s), adverse events, and serious adverse events. The DSMC will meet on a quarterly basis to review this report. Following the review of these quarterly reports, the Committee will recommend to the Co-PIs whether the study should continue, be terminated, or make recommended modifications. In addition, reports will be sent to the Office of Responsible Research Practices and the IRB. In addition, the DSMC will conduct an audit of the grant on a periodic basis and evaluate compliance with adverse events reporting and the data and safety monitoring plan. Their findings will be forwarded to the PI for inclusion in annual reports. The Co-PIs will be responsible for reporting adverse events to the DSMC, IRB, and the Office of Responsible Research Practices within 48 hours of the occurrence. A serious event is defined as one that: is fatal or life-threatening (i.e., results in an immediate risk of death); requires hospitalization; results in persistent or significant injury or disability; or is an important medical event that when based upon appropriate medical judgment, may jeopardize the participant, and may require medical or surgical intervention to prevent one of the outcomes listed above.

**VII. Incentives**

*aim 1*

Participants who complete the MINDBODYSTRONG evaluation will receive $50.

*aim 2*

Nurses who score moderate-to-high will receive $20 for their completion of the baseline questionnaire presented after completing the ISP, $20 for their completion of the 8-week post-survey, $30 for their completion of the 3 month survey, $30 for completion of the 6 month survey and $70 for completion of the 12 month survey.**References**

Brent, D.A. (2009). The treatment of SSRI-resistant depression in adolescents (TORDIA): In search of the best next step. *Depression and Anxiety*, *26*(10), 871–874.

Davidson, J. E., Accardi, R., Sanchez, C., Zisook, S., & Hoffman, L. A. (2020). Sustainability and outcomes of a suicide prevention program for nurses. *Worldviews on Evidence‐Based Nursing*, *17*(1), 24-31.

Davidson, J., Mendis, J., Stuck, A.R., DeMichele, G., & Zisook, S. (2018b). Nurse suicide: Breaking the silence. *NAM Perspectives*. Discussion Paper, National Academy of Medicine, Washington, DC. doi: 10.31478/201801a

Davidson, J.E., Proudfoot, J., Lee, K., Terterian, G., & Zisook, S. (2020) A longitudinal Analysis of Nurse Suicide in the United States (2005-2016). *Worldviews on Evidence-Based Nursing, 17*(1), 6-15. Doi: 10.1111/wvn.12419

Davidson, J.E., Stuck, A.R., Zisook, S., & Proudfoot, J. (2018c). Testing a Strategy to Identify Incidence of Nurse Suicide in the United States. *Journal of Nursing Administration, 48*(5), 259-265. doi: 10.1097/NNA.0000000000000610.

Davidson, J.E., Zisook, S., Kirby, B., DeMichele, G., Norcross, W. (2018). Suicide prevention: A healer education and referral program for nurses. *Journal Of Nursing Administration, 48*(2), 85-92. doi: 10.1097/NNA.0000000000000582.

Dolan, E.D., Mohr, D., Lempa, M., Joos, S., Fihn, S.D., Nelson, K.M. & Helfrich, C.D. (2015). Using a single item to measure burnout in primary care staff: a psychometric evaluation. *Journal of General Internal Medicine, 30*(5), 582-587. doi: 10.1007/s11606-014-3112-6.

Downs, N., Feng, W., Kirby, B., McGuire, T., Moutier, C., Norcross, W., Norman, M., Young, I., & Zisook, S. (2014). Listening to depression and suicide risk in medical students: the Healer Education Assessment and Referral (HEAR) Program. *Academic Psychiatry* : *the journal of the American Association of Directors of Psychiatric Residency Training and the Association for Academic Psychiatry*, *38*(5), 547-553. doi: 10.1007/s40596-014-0115-x.

Holmes, T. H., & Rahe, R. H. (1967). The Social Readjustment Rating Scale. Journal of psychosomatic research, 11(2), 213–218. https://doi.org/10.1016/0022-3999(67)90010-4

Kroenke, K., Spitzer, R.L., & Williams, J.B. (2003). The Patient Health Questionnaire-2: Validity of a two-item depression screener. *Medical Care, 41*(11), 1284-92. DOI: 10.1097/01.MLR.0000093487.78664.3C

Liu, Y., Wu, L.M., Chou, P.L., Chen, M.H., Yang, L.C., & Hsu, H.T. (2016). The influence of work-related fatigue, work conditions, and personal characteristics on intent to leave among new nurses. *Journal of Nursing Scholarship, 48*(1), 66-73. doi: 10.1111/jnu.12181.

Löwe, B., Decker, O., Müller, S., Brähler, E., Scheilberg, D., Herzog, W., & Herzberg, P.Y. (2008). Validation and standardization of the Generalized Anxiety Disorder Screener (GAD-7) in the general population. *Medical Care, 46*(3), 266-274. doi: 10.1097/MLR.0b013e318160d093.

Martinez, S., Tal, I., Norcross, W., Newton, I.G., Downs, N., Seay, K., McGuire, T., Kirby, B., Chidley, B., Tiamson-Kassab, M., Lee, D., Hadley, A., Doran, N., Jong, P., Lee, K., Moutier, C., Norman, M. & Zisook, S. (2016). Alcohol use in an academic medical school Environment: A UC San Diego healer education assessment and referral (HEAR) report. *Annals of Clinical Psychiatry, 28*(2), 85-94.

Melnyk, B.M., Jacobson, D., Kelly, S., Belyea, M., Shaibi, G., Small, L., O’Haver, J., & Marsiglia, F.F. (2013a). Promoting healthy lifestyles in high school adolescents: A randomized controlled trial. *American Journal of Preventive Medicine*, *45*(4), 407-415. dx.doi.org/10.1016/j.amepre.2013.05.013

Mortali, M., & Moutier, C. (2018). Facilitating help-seeking behavior among medical trainees and physicians using the Interactive Screening Program. *Journal of Medical Regulation*, *104*(2), 27-36.

Moutier, C., Norcross, W., Jong, P., Norman, M., Kirby, B., McGuire, T., & Zisook, S. (2012). The suicide prevention and depression awareness program at the University of California, San Diego School of Medicine*. Academic Medicine: Journal of the Association of American Medical Colleges*, *87*(3):320-326. doi: 10.1097/ACM.0b013e31824451ad

Mundt, J.C ., Greist, J.H., Gelenberg, A.J., Katzelnick, D.J., Jefferson, J.W., Modell, J.G. (2010). Feasibility and validation of a computer-automated Columbia–Suicide Severity Rating Scale using interactive voice response technology*. Journal of Psychiatric Research, 44*(16), 1224–1228.

National Academy of Medicine. (December 15, 2016). Action collaborative to promote clinician well-being and combat burnout, depression and suicide among healthcare workers. https://nam.edu/national-academy-of-medicine-launches-action-collaborative-to-promote-clinician-well-being-and-combat-burnout-depression-and-suicide-among-health-care-workers/. Accessed June 1, 2019.

Ng, Q. X., De Deyn, M., Lim, D. Y., Chan, H. W., & Yeo, W. S. (2020). The wounded healer: A narrative review of the mental health effects of the COVID-19 pandemic on healthcare workers. *Asian journal of psychiatry*, *54*, 102258. Advance online publication. doi: 10.1016/j.ajp.2020.102258

Norcross, W.A., Moutier, C., Tiamson-Kassab, M., Jong, P., Davidson, J.E., Lee, K.C., Newton, I.G., Downs, N.S., & Zisook, S. (2018). Update on the UC San Diego Healer Education Assessment and Referral (HEAR) Program. Journal of Medical Regulation, 104(2),17-26. doi.org/10.30770/2572-1852-104.2.17

Posner, K., Brown, G.K, Stanley, B., Brent, D.A., Yershova, K.V., Oquendo, M.A., … Mann, J.J. (2011). The Columbia-Suicide Severity Rating Scale: Initial validity and internal consistency findings from three multisite studies with adolescents and adults. *American Journal of Psychiatry, 168(12)*, 1266-1277.

Pospos, S., Tal, I., Iglewicz, A., Newton, I..G., Tai-Seale, M., Downs, N., Jong, P., Lee, D., Davidson, J.E., Lee, S.Y., Rubanovich, C.K., Ho, E.V., Sanchez, C., & Zisook, S. (2019). Gender differences among medical students, house staff, and faculty physicians at high risk for suicide: A HEAR report. *Depression and Anxiety, 36*(10), 902-920. doi: 10.1002/da.22909.

Price, J.L. & Mueller, C.W. (1983). Professional turnover: The case of nurses. *Journal for Continuing Education Professionals in Health Sciences, 3*(2), 97-99. doi.org/10.1002/chp.4760030220

Rahman, A., & Plummer, V. (2020). COVID-19 related suicide among hospital nurses; case study evidence from worldwide media reports. *Psychiatry research*, *291*, 113272.

Reger, M. A., Stanley, I. H., & Joiner, T. E. (2020). Suicide Mortality and Coronavirus Disease 2019-A Perfect Storm?. *JAMA psychiatry*, 10.1001/jamapsychiatry.2020.1060. Advance online publication. doi: 10.1001/jamapsychiatry.2020.1060

Sampson, M., Melnyk, B.M., Hoying, J. (2019). Intervention effects of the MINDBODYSTRONG cognitive behavioral skills building program on newly licensed registered nurses' mental health, healthy lifestyle behaviors, and job satisfaction. Journal of Nursing Administration; 49(10), 487-495. doi: 10.1097/NNA.0000000000000792.

Sampson, M., Melnyk, B.M., & Hoying, J. (2020). The MINDBODYSTRONG Intervention for New Nurse Residents: 6 Month Effects on Mental Health Outcomes, Healthy Lifestyle Behaviors and Job Satisfaction. *Worldviews on Evidence-Based Nursing.*

Stavarski, D.H., Millsaps, U., Pumariega, A.J., Posner, K. , Romig, B., … Castellucci, M.J. (2011). Suicide screening in a general hospital setting: Initial results. *Proceedings of the Eastern Nursing Research Society, 23^rd^ Annual Scientific Sessions, Philadelphia, PA, p178*.

Zisook, S., Young, I., Doran, N., Downs, M., Hadley, A., Kirby, B., McGuire, T., Moutier, C., Norcross, W., & Tiamson-Kassab, M. (2015). Suicidal Ideation Among Students and Physicians at a US Medical School A Healer Education, Assessment and Referral (HEAR) Program Report. *OMEGA-Journal of Death and Dying, 74*(1), 35-61. doi.org/10.1177/0030222815598045
