# Supplementary material for: A study protocol for the modified interactive screening program plus MINDBODYSTRONG© RCT: A mental health resiliency intervention for nurses
Source: PLoS One. 2024 Jun 6;19(6):e0303425. doi: 10.1371/journal.pone.0303425 (PMC11156330; doi:10.1371/journal.pone.0303425)
Supplement: S3 File — (DOC) [file pone.0303425.s003.doc]

**S3 The Ohio State University Consent to Participate in Research**

| **Study Title:** Modified Interactive Screening Program plus MINDBODYSTRONG: A Mental Health Resiliency Intervention for Nurses |  |
| --- | --- |
| **Researcher:** Bernadette Melnyk, PhD, APRN-CNP, FAANP, FNAP, FAAN |  |
| **Sponsor:** American Foundation for Suicide Prevention |  |

**This is a consent form for research participation.** It contains important information about this study and what to expect if you decide to participate.

**Your participation is voluntary.**

Please consider the information carefully. Feel free to ask questions before making your decision whether or not to participate.

**Purpose:** Determine the effects of the Modified Interactive Screening Program (mISP) screening and referral program combined with an on-line version of the MINDBODYSTRONG© cognitive-behavioral skills building program versus the mISP program alone on nurse depression, suicidal ideation, burnout, anxiety, healthy lifestyle beliefs, healthy lifestyle behaviors and job satisfaction.

**Procedures/Tasks:** If you decide to participate in this study, you will be directed to the American Foundation for Suicide Prevention’s modified interactive screening program (mISP) for risk of suicide. The platform itself is anonymous. Information collected in the mISP is stored differently than information collected throughout the rest of the study. We will not be able to identify any responses provided in the mISP to a specific individual, and you are not required to answer all the questions. You have the choice to provide contact information within the mISP for the counselor to use, such as your phone number and name, to connect. You may also remain anonymous on the mISP platform and communicate entirely through their encrypted service.

The categories of risk on the mISP assessment include depression, suicidal ideation, burnout and anxiety. If you are identified as moderate to high risk, a licensed mental health counselor will send a message and offer support. Depending on your risk, the message will be sent within 24-48 hours.

The counselor will offer support online through an encryption service. How you interact with the counselor is entirely up to you. You may choose to provide an email to receive messages, provide your name and telephone number to contact the counselor, or you can communicate entirely through the mISP encrypted platform, which is anonymous. We are not able to link responses provided in the mISP to any of the information provided in the REDCap surveys. In general, the counselor is to encourage help-seeking, whether via the phone (if you choose to disclose your phone number) or through the encrypted online dialogue system. If you warrant continued mental health treatment, you will be supported through the process of one or two additional sessions with the while connecting with a provider in your area if further treatment is requested. Given the anonymous and conversational nature of this interaction, the level (duration and frequency) of engagement is dependent upon the participant.

If you are **not** identified as having moderate to severe risk of suicide, you will be thanked for their time after completing the mISP questionnaire. A counselor will reach out and provide you with non-study resources.

If you **are** identified as moderate to high risk, as indicated by the mISP, you will be eligible to continue study participation. You will be directed back to the REDCap platform and asked to complete additional confidential questionnaires related to depression, suicidal ideation, burnout, anxiety, life events, healthy lifestyle beliefs, healthy lifestyle behaviors and job satisfaction. Some of these questions may repeat, or be similar to, the questions asked in the mISP. This repetition occurs once during the study. As the mISP is completely anonymous, it is necessary for the quality of the research to ask some questions again confidentially. Once these surveys are complete, eligible participants will be randomly assigned to receive the MINDBODYSTRONG© program or continue with mISP alone. All participants, in addition to surveys completed at baseline, will be contacted by email to repeat survey measures at eight weeks, three months, six months and 12 months. You will also receive study reminder messages at month five and month nine.

We use multiple systems to ask questions and communicate during this study. The American Foundation for Suicide Prevention’s mISP is designed to be anonymous, but you can add information that would identify you. The information provided in the mISP is not linked to any information provided in other parts of the study. This can be confusing. For this reason, we will attempt to call you by phone if you decide to participate in the study. This is only to ensure your questions about the study have been answered. For this reason, we will ask for your phone number with your name at the bottom of this form. Please refer to the table below for clarity regarding the different programs we use in this study:

|  | **Data Collection** |
| --- | --- |
| **REDCap Consent** | Confidential |
| **mISP** | Anonymous and not connected to other study data.  You can provide confidential contact information for use communicating with the counselor, which would make your use of the platform confidential and not anonymous.  mISP information is not connected to the remaining study data collection. |
| **Study Interactions After Screening** |  |
| **REDCap surveys** | Confidential |
| **eLearning Platform for MINDBODYSTRONG participants** | Confidential |

**MINDBODYSTRONG© program**

If assigned to the MINDBODYSTRONG© program you will receive 7- online interactive sessions delivered weekly, with an additional accompanying session that addresses coping with trauma. Skills building activities are provided for independent completion between sessions. Reminders will be sent weekly to complete the next MINDBODYSTRONG© session via the online platform where MINDBODYSTRONG sessions will be delivered. A MINDBODYSTRONG© facilitator will check in with you by phone at baseline, weeks 3 and 5 of the on-line program to reinforce key program concepts and assess your progress.

Because of the need to contact you during the study period, your name, phone number, and email address are required. All other questions are voluntary. You may skip one or more questions and continue with the program.

**Duration:**

The mISP questionnaire takes approximately 10 minutes to complete. If you warrant continued mental health treatment, you will be supported through the process of one or two additional sessions with the counselor and then connected with a provider in their area if further treatment is requested. Given the conversational nature of this interaction, the level (duration and frequency) of engagement is dependent upon the participant.

If assigned to the MINDBODYSTRONG© program you will receive 7- online interactive sessions delivered weekly, with an additional accompanying session that addresses coping with trauma. The total time commitment for the MINDBODYSTRONG© program is 8 weeks and each session is approximately 45 minutes, including time to complete skills building activities and facilitator discussions.

Additionally, all participants will be asked to complete an online survey administered at baseline, 8 weeks, three months, six months, and 12 months each survey will take about 15 minutes to complete. In total the MINDBODYSTRONG program will take about 9.5 hours to complete.

You may leave the study at any time. If you decide to stop participating in the study, there will be no penalty to you, and you will not lose any benefits to which you are otherwise entitled. Your decision will not affect your future relationship with The Ohio State University, Healthy Nurse Healthy Nation, the American Nurses Association (ANA) or the American Foundation for Suicide Prevention.

**Risks and Benefits:** Data will be collected from you and stored online, so there is a potential risk of a data breach that would compromise the confidentiality of your responses. There may be no direct benefits to you from your participation, although we hope that you will learn valuable information about your personal health and wellness. We anticipate that the study may generate knowledge and scientific data that will benefit nurses in the future, and by validating these programs and procedures for future use. If you are identified as having moderate to severe risk of suicide you will be contacted via the encrypted platform within 24 hours; however, it is not guaranteed that a counselor would be able to address any imminent suicide risk before then. Please note that this is not a crisis intervention service, and that no follow-up services will be provided unless requested.

# Confidentiality:

We will work to make sure that no one sees your online responses without approval. But, because we are using the Internet, there is a chance that someone could access your online responses without permission. In some cases, this information could be used to identify you.

Also, there may be circumstances where this information must be released. For example, personal information regarding your participation in this study may be disclosed if required by state law. Also, your records may be reviewed by the following groups (as applicable to the research):

- Office for Human Research Protections or other federal, state, or international regulatory agencies;
- The Ohio State University Institutional Review Board or Office of Responsible Research Practices;
- The sponsor, if any, or agency (including the Food and Drug Administration for FDA-regulated research) supporting the study.
- IRB approved study team members at the University of California San Diego

**Future Research:** Your de-identified information may be used or shared with other researchers without your additional informed consent.

**Incentives:**

By law, payments to participants are considered taxable income. Nurses who score moderate-to-high will receive $20 for their completion of the baseline questionnaire presented after completing the ISP, $20 for their completion of the 8-week post-survey, $30 for their completion of the 3 month survey, $30 for completion of the 6 month survey and $70 for completion of the 12 month survey.

# Participant Rights:

You may refuse to participate in this study without penalty or loss of benefits to which you are otherwise entitled. If you are a student or employee at Ohio State, your decision will not affect your grades or employment status.

If you choose to participate in the study, you may discontinue participation at any time without penalty or loss of benefits. By agreeing to participate, you do not give up any personal legal rights you may have as a participant in this study.

An Institutional Review Board responsible for human subjects research at The Ohio State University reviewed this research project and found it to be acceptable, according to applicable state and federal regulations and University policies designed to protect the rights and welfare of participants in research.

Dr. Bernadette Melnyk, the person responsible for this study, could financially benefit from testing the MINDBODYSTRONG program. A conflict of interest committee at Ohio State has reviewed this information and determined that Dr. Melnyk’s involvement presents no additional significant risk to the study's participants. Any questions about this information can be answered by Dr. Jacqueline Hoying (614-359-2515).

# Contacts and Questions:

For questions, concerns, or complaints about the study, or you feel you have been harmed as a result of study participation, you may contact Bernadette Melnyk, PhD, APRN-CNP, FAANP, FNAP, FAAN at, 614-292-4844.

For questions about your rights as a participant in this study or to discuss other study-related concerns or complaints with someone who is not part of the research team, you may contact the Office of Responsible Research Practices at 1-800-678-6251 or [hsconcerns@osu.edu](mailto:hsconcerns@osu.edu).

# Providing consent

I have read (or someone has read to me) this page and I am aware that I am being asked to participate in a research study. I have had the opportunity to ask questions and have had them answered to my satisfaction. I voluntarily agree to participate in this study. I am not giving up any legal rights by agreeing to participate.

To print or save a copy of this page, select the print button on your web browser.

**I verify that I am a licensed nurse Y/N**

**I verify that I am aged 18 or older Y/N**

**I verify that I have not participated in the MINDBODYSTRONG© program previously Y/N**

**I verify that I have not previously participated in this study, the MentalWellbeing4Nurses study, previously Y/N**

**Please click the button below to proceed and participate in this study. If you do not wish to participate, please close out your browser window.**

Name: _______________________________

Date: __________________ Time: ________________

Email Address: ________________________________

Telephone Number: _____________________________
