# Supplementary material for: A study protocol for the modified interactive screening program plus MINDBODYSTRONG© RCT: A mental health resiliency intervention for nurses
Source: PLoS One. 2024 Jun 6;19(6):e0303425. doi: 10.1371/journal.pone.0303425 (PMC11156330; doi:10.1371/journal.pone.0303425)
Supplement: S4 File — (DOCX) [file pone.0303425.s004.docx]

**S4 MINDBODYSTRONG^©^ Program Evaluation Questionnaire**

What things or skills that you learned in the digitized MINDBODYSTRONG program do you plan to continue to use?
