# Supplementary material for: A study protocol for the modified interactive screening program plus MINDBODYSTRONG© RCT: A mental health resiliency intervention for nurses
Source: PLoS One. 2024 Jun 6;19(6):e0303425. doi: 10.1371/journal.pone.0303425 (PMC11156330; doi:10.1371/journal.pone.0303425)
Supplement: S5 File — (DOCX) [file pone.0303425.s005.docx]

**S5 Modified Interactive Screening Program Evaluation Questionnaire**

Please answer the following questions about the modified interactive screening program (mISP). This program includes the screening questions and interactions that take place with the counselor through the MentalWellbeing4Nurses website.

1) Did you find the mISP helpful? ___Yes ____No

2) If you found the mISP helpful, in what ways did it help you?

3) What would you change about or add to the mISP?

4) Did you interact with the mISP counselor directly? __Yes __No

5) If you interacted directly with the mISP counselor, what was most helpful to you?

6) Have you received any mental health treatment during the study? __Yes __No

7)If yes, what treatment have you received?

__ counseling

__ medication

__other

8) If yes, when did you start treatment?

__ prior to the study

__ after referral from the mISP counselor

__some other time

9)If yes, are you currently receiving treatment? Select all that apply.

__none

__counseling

__medication

__other

Thanks so much for completing this evaluation of the mISP!
