# Supplementary material for: A study protocol for the modified interactive screening program plus MINDBODYSTRONG© RCT: A mental health resiliency intervention for nurses
Source: PLoS One. 2024 Jun 6;19(6):e0303425. doi: 10.1371/journal.pone.0303425 (PMC11156330; doi:10.1371/journal.pone.0303425)

S6 MINDBODYSTRONG^©^ Facilitator Responses

Session Contact Log

Please complete the survey below. Thank you!

Contact Timepoint Baseline

Week 3


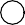

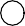

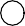


Week 5

Baseline Script

Hello, my name is [NAME HERE] and today we will be meeting briefly to discuss the program in which you are about to begin participating. Do you have any questions regarding accessing the program? It is housed in an e-Learning platform to which you have been/will be given access.

MINDBODYSTRONG is a 7 -session program with an optional additional session created to help you learn skills to think more positively and feel better emotionally. The MINDBODYSTRONG program approaches mental health and wellbeing in two ways: 1) it provides techniques that research on cognitive-behavioral therapy has found to reduce stress, anxiety and depressive symptoms, and 2) it builds resiliency as well as coping and problem-solving skills.

It is important that you work through one session every week. However, if you happen to skip a week, pick up where you left off and do not jump ahead or complete sessions out of order as each session's content builds upon the last session. For each session, there are videos that present the content, followed by various skills building activities to practice. The skills building activities can be downloaded and printed ahead of time. I suggest keeping all of them together in a folder. Practicing these skills building activities is key to creating new thinking and behaving patterns as well as healthy habits.

My role is to be a resource for you to answer your questions and also to follow-up with you twice during your participation in MINDBODYSTRONG, ideally after the third and fifth sessions. Can we schedule the first follow-up now? What days and times are best for you? You can plan to meet with me for about 10 to 15 minutes for each of these follow-up times.

The program is meant to be self-paced so that you can spend as much time on a session as you want. We recommend you complete one session each week. You will receive an email reminder once a week to complete sessions. If you would like, we can also send you a text message reminder. Would that be helpful? If Y, what number would you like to use for those text messages?

Remember, practicing the skills after each session daily is key to improved mental health and coping outcomes.

Session 1-3 Follow-up

How are you? Have you been able to access the MINDBODYSTRONG course and review the materials? Do you have any issues or questions? Is there anything you would like to share about your experience with the MINDBODYSTRONG program so far?

I'd like to review some of the key concepts with you now. The thinking, feeling, behaving triangle is the core of this program. Recognizing how powerful our thoughts are in influencing our feelings and behaviors was eye opening when I first participated in this program. Our brains are like a computer: the more positive we put into them, the more positive we will feel and behave. In MINDBODYSTRONG, there are several ways to provide positive input: by saying your positive self-statements 10x in the morning and 10x at night, by practicing gratitude, by reading a positive book, and by cognitive reframing, that is, turning automatic negative or unhelpful thoughts into positive ones to feel emotionally better. So the next time that you feel depressed, anxious or angry, stop and say to yourself "What was I just thinking?"; "Is it true or helpful or do I have the evidence to back it up?" The answer will probably be no, so turn that negative or unhelpful thought around to a positive one to feel emotionally better.

Have you been able to identify trigger or activating events that often lead to some of your cognitive distortions or automatic negative unhelpful thoughts? Everyone has cognitive distortions and recognizing them is a key step in being able to reframe them. Cognitive distortions are often triggered by stressful situations.

A great way for us to worry less and feel good is to focus on what is going on in the present moment. Two of our most wasted emotions are worry about the future or guilt about the past. As we stay in the present moment, we can be more effective at what we do and in our relationships. Many activities can help us to be in the present moment and the practice of meditation or imagery is a great way to train our mind to be in the present moment. What being in the present moment activities have you tried? Do you find staying in the present moment is helping you to worry less and feel less stressed?

By now, you have l probably identified a habit or coping strategy that you would like to either start or stop using. What is that for you? Identifying the habit you want to change is the first step in the change process. Next, set the goal that you would like to accomplish. Infuse that goal with positivity by believing you can accomplish it. When you believe, anything is possible. Then take action.

Do you have any questions about MINDBODYSTRONG at this point? In the next sessions, you'll learn about problem solving, goal setting, and dealing with emotions in healthy ways. Remember, it is very important to practice the skills building activities after each session as they help you put what you are learning into daily practice. It often takes 30 to 66 days to make or break a new habit or to change unhelpful or negative thinking. I'd like to meet with you in about 2 weeks, after session five. What day and time works best for you?

Session 4 & 5 Follow-up

Thank you for meeting with me today. How is your participation in MINDBODYSTRONG going? Do you have any questions? Is there anything you'd like to share about the past two weeks and your experience with MINDBODYSTRONG?

Have you noticed a change in your thoughts as you are practicing your positive self-statements? Have you remembered to say those statements aloud every morning and evening? If not, what barriers have you identified and what have you done to overcome those barriers? I know I have a hard time remembering to say them, so I set an alarm on my phone with its own ringtone to remind me.

Today, I'd like to review some of the key concepts in MINDBODYSTRONG from the last two sessions. When we are aware of how we think, feel and behave when we are stressed, it can help us intervene early and implement healthy coping strategies that can reduce stress, such as deep abdominal breathing. Remember, when your mood changes and you feel stressed, anxious or depressed, catch your thoughts. Ask yourself "What was I just thinking?" and check those thoughts by asking "Do I think these thoughts are helpful or true?" and "Do I have the evidence to back them up?" Chances are they are not true, so it's important to change them from negative to positive to feel better.

When we are not able to manage our stress or when we use unhealthy strategies to cope, we often feel anxious, stress or depressed. When people are experiencing these emotions to the point they are interfering with their concentration or functioning, it is important to get help from a mental health professional or primary care provider. If you ever get to that point, it is a strength to recognize when you need help, not a weakness. Always remember the 988 number, the suicide prevention and crisis hotline, as you never know when you or someone you know may need to talk anonymously with a mental health professional.

Setting a goal is the first step in moving toward accomplishing it. Often, as we work towards a goal, barriers arise. A four step process of problem solving was introduced in session 4 that can be used to help overcome both small and large barriers. Remember to identify the problem, list the causes of the problem, and then list possible solutions with their pros and cons. Once that is done, pick the best solution and act on it. Delaying action can often make you feel more anxious.

Finally, let's review using some self-control strategies that can help us feel better emotionally and take control of situations instead of losing control, which will help us to feel better about our self as well as feel better emotionally. Have you identified strategies that you use and find helpful? Some healthy strategies include exercise, relaxation techniques like guided or mental imagery, spending time with friends and pets, or doing hobbies you like. Self-control strategies include positive self-talk, counting to 100 or saying ABCs, deep abdominal breathing, or going for a walk.

Communicating with others effectively can include reflective and active listening, tone of voice, body language, and facial expressions. Engaging with others using "I" statements can help respond to others in a non-judgmental way.

In the last three sessions of MINDBODYSTRONG, you will learn about coping with stressful situations, emotional regulation and sleep. The final session will pull it all together by incorporating elements from each of the MINDBODYSTRONG sessions.

An additional module was added to the MINDBODYSTRONG program to provide a few extra skills to help deal with post-traumatic stress. Even if you are not dealing with post-traumatic stress, you might want to watch the additional module for a few additional helpful strategies. Would you be interested in watching it? Yes/No

Remember, the more you practice the skills taught in each session of MINDBODYSTRONG and they become daily habits, the better you will feel emotionally and be able to deal with challenges that come your way. You can do it!

This is our last scheduled huddle. From here, you will complete the final sessions on your own. You will continue to receive reminders for the remainder of the MINDBODYSTRONG program. You will also receive emailed study surveys at after you complete the program, and again at 3 months, 6 months, and 12 months. We would so appreciate you completing these surveys. After each set of surveys is complete, you will receive an emailed gift card. If you have any questions or concerns throughout the study, please don't hesitate to reach out!

Research Staff Member's Initials

Research Staff Member's Initials

Research Staff Member's Initials

Date and Time of Contact #1 (Baseline)

Date and Time of Contact #1 (Week 3)

Date and Time of Contact #1 (Week 5)

Outcome of Contact #1 (Baseline)

Spoke directly with Participant Phone rang but no answer

Participant answered but was unable to speak Patient answered and hung up

Patient answered and call dropped LVM

Unable to LVM

Phone not accepting calls (i.e., disconnected, out of service, busy signal etc.)

Wrong number Texted

Other

Outcome of Contact #1 (Week 3)

Spoke directly with Participant Phone rang but no answer

Participant answered but was unable to speak Patient answered and hung up

Patient answered and call dropped LVM

Unable to LVM

Phone not accepting calls (i.e., disconnected, out of service, busy signal etc.)

Wrong number Texted

Other

Outcome of Contact #1 (Week 5)

Spoke directly with Participant Phone rang but no answer

Participant answered but was unable to speak Patient answered and hung up

Patient answered and call dropped LVM

Unable to LVM

Phone not accepting calls (i.e., disconnected, out of service, busy signal etc.)

Wrong number Texted

Other

Baseline Other Contact #1 ( please specify)

Week 3 Other Contact #1 ( please specify)

Week 5 Other Contact #1 ( please specify)

Date and Time of Contact #2 (Baseline)

Date and Time of Contact #2 (Week 3)

Date and Time of Contact #2 (Week 5)

Outcome of Contact #2 (Baseline)

Spoke directly with Participant Phone rang but no answer

Participant answered but was unable to speak Patient answered and hung up

Patient answered and call dropped LVM

Unable to LVM

Phone not accepting calls (i.e., disconnected, out of service, busy signal etc.)

Wrong number Texted

Other

Outcome of Contact #2 (Week 3)

Spoke directly with Participant Phone rang but no answer

Participant answered but was unable to speak Patient answered and hung up

Patient answered and call dropped LVM

Unable to LVM

Phone not accepting calls (i.e., disconnected, out of service, busy signal etc.)

Wrong number Texted

Other

Outcome of Contact #2 (Week 5)

Spoke directly with Participant Phone rang but no answer

Participant answered but was unable to speak Patient answered and hung up

Patient answered and call dropped LVM

Unable to LVM

Phone not accepting calls (i.e., disconnected, out of service, busy signal etc.)

Wrong number Texted

Other

Baseline Other Contact #2 ( please specify)

Week 5 Other Contact #2 ( please specify)

Date and Time of Contact #3 (Baseline)

Date and Time of Contact #3 (Week 3)

Date and Time of Contact #3 (Week 5)

Outcome of Contact #3 (Baseline)

Spoke directly with Participant Phone rang but no answer

Participant answered but was unable to speak Patient answered and hung up

Patient answered and call dropped LVM

Unable to LVM

Phone not accepting calls (i.e., disconnected, out of service, busy signal etc.)

Wrong number Texted

Other

Outcome of Contact #3 (Week 3)

Spoke directly with Participant Phone rang but no answer

Participant answered but was unable to speak Patient answered and hung up

Patient answered and call dropped LVM

Unable to LVM

Phone not accepting calls (i.e., disconnected, out of service, busy signal etc.)

Wrong number Texted

Other

Outcome of Contact #3 (Week 5)

Spoke directly with Participant Phone rang but no answer

Participant answered but was unable to speak Patient answered and hung up

Patient answered and call dropped LVM

Unable to LVM

Phone not accepting calls (i.e., disconnected, out of service, busy signal etc.)

Wrong number Texted

Other

Baseline Contact #3 Other ( please specify)

Week 3 Other Contact #3 ( please specify)

Week 5 Other Contact #3 ( please specify)

Week 3 Intended Follow-Up (Day and Time)

Week 5 Intended Follow-Up (Day and Time)

Did the participant complete all required sessions Yes

since the last meeting? No


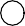

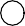

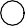


Not Applicable ( If Baseline)


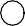

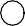


You will receive MINDBODYSTRONG reminders via email. Yes Would you like to receive text reminders about No MINDBODYSTRONG in addition to email reminders.

What number would you like to use for the text message reminders?

Given the participant did not complete all required sessions what was their reason for incompletion ( if any)?

Sessions Completed by Participant ( since last meeting)

Session 1

Session 2

Session 3

Session 4

Session 5

Session 6

Session 7

Session 8

Do you have questions regarding accessing the program?

Have you been able to access the MINDBODYSTRONG course and review the materials?

Do you have any questions or issues with the program or skills building activities?

Is there anything you would like to share about your experience with the MINDBODYSTRONG program so far?

Have you been able to identify trigger events that often lead to some of your cognitive distortions or automatic negative unhelpful thoughts?

Which "being in the present moment activities" have you tried?

Do you find staying in the present moment is helping you to worry less and feel less stressed?

By now, you have l probably identified a habit or coping strategy that you would like to either begin to use or stop using. What is that for you?

Do you have any questions about MINDBODYSTRONG at this point?

How is your participation in MINDBODYSTRONG going?

Is there anything you'd like to share about the past

two weeks and your experience with MINDBODYSTRONG?

Have you noticed a change in your thoughts as you are practicing your positive self-statements?

Have you remembered to say those statements aloud every morning and evening?

If not, what barriers have you identified and what have you done to overcome those barriers?

Have you identified strategies that you use and find helpful?

Would you be interested in watching the 8th module? Yes No

FACILITATOR FEEDBACK: How did the facilitator session go with the participant? Please provide specific information if possible.


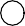

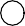

Supplement: S6 File — (DOCX) [file pone.0303425.s006.docx]
